# 

**Published:** 1872-03

**Authors:** 


### OHIO COLLEGE OF DENTAL SURGERY.

### SPRING COURSE.

The Spring course in this Institution will commence on the 10th of April, prox , and continue till the 15th day of June.

This course is organized with special reference to teaching the practical departments, and especially Operative Dentistry; of affording to all students who wish to progress most rapidly, and make the best use of their time, an opportunity to do so. It will also give an excellent opportunity to those already in practice, to post up on the advance methods of operating, and treating diseases of the teeth and mouth. Some have already signified their intention to be present, at least part of the time, during this course.

There will be one lecture each day upon principles. The afternoon will be wholly devoted to operating and demonstrations, under the direction and supervision of competent teachers, prominent among whom will be Professor C. Palmer, than whom our profession can boast of no superior as an operator or teacher; he is so well known, however, that this does not need our statement. All aids for demonstration will be brought into requisition.

The lectures delivered during the course upon Anatomy, Physiology, Pathology, &c., will have a more direct bearing upon practice than those of the Winter term. They will, however, be given for the most part by the same teachers, viz:

C. KEARNES.—Anatomy and Surgery of the head and face.
E. RIVES.—Physiology and Microscopy.
F. BRENNING.—Pathology, and treatment of the mouth and contiguous parts.
J. S. CASSIDY—Chemistry in its application to Dental practice.
J. TAFT.—Principles of Dental Hygiene and Operative Dentistry.
C. PALMER, WM. TAFT, H. R. SMITH, H. A. SMITH.—Practical department.

Text books same as for Winter course.

Ticket for the course, $30, to be paid on entering.

J. TAFT, DEAN, 117 W. 4th St.

It will be observed that this course offers great advantages for practical instruction in operating, as the time will be largely occupied in this work; with much longer days and a far better light, and more pleasant weather than in the winter season.

Let all who intend to enter the class give notice of the fact at the earliest moment possible, and then be here as near the 10th of April as convenient. There will be nothing to hinder practitioners from coming in for a short time, when it is impracticable for them to leave home for the full term. The aim is to give valuable practicable instruction, and not to make it to any extent take the place of the regular course, but to be simply additional to it.

Good board can be had for much less than in the winter; and it is a far more pleasant time to be in the city.

All who have instruments for operating are requested to bring them.

### The Dental Register,

### OF THE WEST.

A Monthly Magazine, devoted entirely to the interest of the Dental Profession.

**Terms $3.00 per Year. Single Nos. 25 Cents.**

EDITED BY PROF. J. TAFT,

And Published by SPENCER & MOORE of the Ohio Dental Depot.

The volume commences in January and closes in December.

The Register being issued monthly is always fresh therefore indispensable to the practitioner who desires to keep posted up with the new inventions and improvements which are daily developed. Neither expense nor effort will be spared to give value and variety to its contents. Articles will be illustrated with well executed engravings whenever they will add to the interest or assist in explanation.

Its extensive circulation and frequency of issue makes it one of the BEST DENTAL ADVERTISING MEDIUMS in the United States.

RATES OF ADVERTISING.

One page, one year, $50. Half page, one year. $30. Quarter page, one year, $20.

All advertising bills payable in advance, or at furthest after the issue of the first number containing the advertisement. All transient advertisements to be paid for in advance.

☞CONTRIBUTIONS SOLICITED.

Exclusively Editorial Communications should be addressed to

**Dr. J. TAFT, Editor.**

The latest number of the Register may be ordered with or without other goods at any time, and all letters should be addressed to DENTAL REGISTER,

